# Tools for teen moms to reduce infant obesity: a randomized clinical trial

**DOI:** 10.1186/s12889-015-1345-x

**Published:** 2015-01-21

**Authors:** Mildred A Horodynski, Kami Silk, Gary Hsieh, Alice Hoffman, Mackenzie Robson

**Affiliations:** College of Nursing, Michigan State University, 1355 Bogue Street, Bott Nursing Building, East Lansing, MI 48824 USA; Department of Communication, Michigan State University, 404 Wilson Road, East Lansing, MI 48824 USA; Department of Human Centered Design and Engineering, University of Washington, 414 Sieg Hall, Seattle, WA 98195 USA

**Keywords:** Infant, Obesity, Social media, Technology, Low-income population, Intervention

## Abstract

**Background:**

Unhealthy infant feeding practices, such as a combination of formula feeding and early introduction of solids may lead to rapid or excessive weight gain in early infancy. Adolescent mothers’ feeding behaviors are most directly related to infant weight gain in the first year of life. Compared to adult mothers, adolescent mothers are less knowledgeable, less responsive, more controlling, and less skilled in infant feeding, which interferes with infants’ healthy growth. The Tools for Teen Moms trial aims to compare the effect of a social media intervention for low-income adolescent, first-time mothers of infants 2 months of age or younger, versus standard care on infant weight, maternal responsiveness, and feeding style and practices. The intervention is conducted during the infant’s first four months of life to promote healthy transition to solids during their first year. Tools for Teen Moms is an intervention delivered via a social media platform that actively engages and coaches low-income adolescent mothers in infant-centered feeding to reduce rapid/excessive infant weight gain in the first six months of life.

**Methods/Design:**

We describe our study protocol for a randomized control trial with an anticipated sample of 100 low-income African- American and Caucasian adolescent, first–time mothers of infants. Participants are recruited through Maternal-Infant Health Programs in four counties in Michigan, USA. Participants are randomly assigned to the intervention or the control group. The intervention provides infant feeding information to mothers via a web-based application, and includes daily behavioral challenges, text message reminders, discussion forums, and website information as a comprehensive social media strategy over 6 weeks. Participants continue to receive usual care during the intervention. Main maternal outcomes include: (a) maternal responsiveness, (b) feeding style, and (c) feeding practices. The primary infant outcome is infant weight. Data collection occurs at baseline, and when the baby is 3 and 6 months old.

**Discussion:**

Expected outcomes will address the effectiveness of the social media intervention in helping teen mothers develop healthy infant feeding practices that contribute to reducing the risk of early onset childhood obesity.

**Trial registration:**

Clinical Trials.Gov NCT02244424, June 24, 2014

## Background

Rapid weight gain (RWG) during infancy is one of the strongest risk factors for obesity later in childhood [1]. A growing body of evidence shows the incidence of infant obesity, especially in low-income infants, is rapidly increasing [2-6]. The first year of life is a critical period for reducing risk of obesity [7-12], particularly the first six months [13,14]. Rapid weight gain in the first six months of life is associated with a sharply increased risk of obesity later in life [15-18] and subsequent health consequences [19]. Unhealthy infant feeding practices may lead to rapid or excessive weight gain in early infancy [12,14,20] and a combination of formula feeding and early introduction of solids contribute to rapid/excessive weight gain [21-25]. Factors associated with rapid/excessive weight gain include socioeconomic status, maternal age, and infant feeding practices [20,26], which include formula feeding, age of introduction to solids, maternal responsiveness, and overfeeding [1,27-32]. Unhealthy mother-infant feeding practices contribute to rapid/excessive infant weight gain [33,34]. Adolescent mothers’ feeding behaviors are most directly related to infant weight gain in the first year of life [31,35,36]. Compared to adult mothers, adolescent mothers are less knowledgeable [37], less responsive, more controlling, and less skilled in infant feeding [38], which interferes with infants’ self-regulation, natural weight trajectory, and healthy growth during the first year of life [39], putting these infants at higher risk for developing obesity [40]. Low-income, adolescent, first-time mothers are also less likely to engage in infant-centered feeding (shared regulation of feeding within the mother-infant dyad) characterized by maternal responsiveness [41] (MR) (positive maternal recognition and responses to infant cues), positive feeding styles [42] (FS) (maternal guided approach to infant feeding), and healthy feeding practices (FP) [43] as recommended by the American Academy of Pediatrics (AAP) [44]. Infant-centered feeding is needed to reduce rapid/excessive gain in the first six months of life [45]. Infant-centered feeding fosters infant feeding self-regulation [46-49], which is associated with healthy growth (weight and length) and is crucial in reducing childhood obesity risk [50-52] and adverse health conditions later in life [53-56].

Few published intervention studies on the prevention or reduction of obesity in infants exist [57]. Practical early intervention strategies are needed to promote infant-centered feeding among adolescent mothers to prevent rapid/excessive infant weight gain in the first six months of life. The few studies published about the efficacy of interventions for obesity prevention in early infancy report mixed results [30,33,58-61]. One home-based study with primarily breastfed babies supported healthy infant growth through delay of introduction to solid foods [58], while a pilot education program with high income and educated participants (which consisted of five pediatrician messages and four coaching sessions by health educators) indicated a delay of solid foods with a trend in lower change in weight-for-length z-scores [57,59]. A double-blind, randomized educational intervention with one session on recognizing satiety cues and limiting volume of formula found no differences on weight gain, formula intake, or parental behavior [30]. Several other studies are in progress [33,61-67], but do not focus on adolescent mothers. This study proposes a highly accessible solution that addresses the unmet knowledge needs of adolescent mothers via an intervention (Tools for Teen Moms) (T4TM) to improve infant-centered feeding (MR, FS, FP) and infant growth. The proposed intervention addresses a deficit in the literature on infant-centered feeding to reduce rapid/excessive infant weight gain that exists for this high-risk population. The purpose of the study is to test a new social media intervention (T4TM) designed by the investigators. The intervention includes an educational web-application, accessible both via computers and smartphones, designed to increase infant-centered feeding through daily behavioral challenge activities, as well as additional resources related to infancy and motherhood, a discussion forum, and a messaging system for asking questions of a registered nurse. Participants receive daily cell phone text message reminders to log on to the website and complete that day’s challenge.

The ubiquity of technology in adolescents’ lives requires new pedagogical methods for interventions to reduce the risk of infant obesity that are adapted to the learning and information-seeking styles preferred by this population [68,69]. There is a dearth of outcome research on nutrition eHealth interventions targeting adolescents [68]. The ability to use technology to deliver interventions is evident: 77% of 12 to 17-year-olds own a cell phone [70-72]. During a six month study, adolescent mothers with infants under six months of age reported seeking health information and social support from online communities, posting 16,670 times, and spending an average of 102.25 hours on these websites [73]. The Pew Survey [71] reports teens regularly go on-line (94%), indicating this age group embraces technology and social media in their everyday lives [74,75]. Social media transcends space and time constraints [76], making health messages more accessible and enabling users to decide when, where, and how they want to receive information. Social media interventions have the ability to facilitate social connections and foster emotional support between adolescent mothers who may feel isolated or disconnected; however, there is a paucity of rigorously designed randomized controlled trials (RCT) of technology-based interventions in health care [77].

T4TM is the first targeted intervention delivered via a social media web-application that actively engages and coaches low-income adolescent mothers in infant-centered feeding to reduce rapid/excessive infant weight gain in the first six months of life. Our study is innovative in multiple ways: 1) it fills a significant gap in obesity research on infant-centered feeding related to MR, FS, and FP together and their contribution in infant accelerated growth and obesity [78]; 2) it promotes daily maternal behavior modification across six weeks for early obesity prevention [59,79]; and 3) it targets an understudied, vulnerable population (low-income, adolescent mothers) [80]. Tools for Teen Moms goes beyond Text4Baby [81], a website that provides infant feeding information to mothers via text messaging. Tools for Teen Moms includes daily behavioral challenges, text message reminders, discussion forums, and a repository of information and references as a comprehensive social media strategy. The design of T4TM differs in its use of: 1) persuasive technology guidelines for health behavior change; 2) challenges to intrinsically motivate participants [82] and support self-efficacy [83]; 3) public commitment and normative influence via social media streams [84]; and 4) inexpensive, easily integrated infant-centered feeding activities [82,83]. The novelty of T4TM is not only its design but also in its ability to affect one of the few potentially modifiable infant obesity risk factors [85-87]. Tools for Teen Moms expands on existing programs focused solely on nutrition via three key techniques: 1) interpreting and responding to infant cues while transitioning to solid foods; 2) providing an attentive and infant-centered authoritative feeding style; and 3) building skills to develop, implement, and sustain an infant-centered feeding plan.

### Aims and hypotheses

The aim of this study is to compare the effect of a social media intervention for low-income adolescent, first-time mothers of infants 2 months of age or younger, versus standard care on infant weight, maternal responsiveness, and feeding style and practices. Adolescent mothers with infants 2 months of age or younger will be assigned randomly to a control/usual care or intervention condition.

The usual Maternal Infant Health Program (MIHP) care only condition consists of voluntary home visits (one week postpartum, six weeks, and six months) and on-going as needed visits (up to nine during the infant’s first year of life) provided by a Registered Nurse (RN), Licensed Social Worker (LSW), Registered Dietician (RD), infant mental health specialist and/or paraprofessional. Maternal Infant Health Program interventions are based on participants’ individualized plans of care following in-home screening assessments. Content includes a flexible plan of care with visits based on identified domains for both the mother (e.g., family planning, transportation, housing, and breastfeeding support), and the infant (e.g., monitoring infant growth). For the T4TM plus usual MIHP care condition, participants get the same usual care treatment as the control group and also receive text messages for six weeks, reminding them to view the T4TM web-application and complete the behavioral challenges. Daily challenges cycle through a pre-determined schedule and focus on: 1) maternal-infant feeding interaction (maternal responsiveness, infant temperament, feeding style); and 2) feeding practices (e.g., delay of early introduction to solids; how much to feed babies) including the feeding environment (e.g., communication with baby, sitting down to eat, turning off the television). Participants also have the option to retroactively complete challenges posted in the last week. We hypothesize:

H1: The T4TM plus MIHP (intervention group) as compared to the MIHP only (control group) will exhibit a greater proportion of infants: a) with a growth in weight that falls below the 85th percentile of the WHO (World Health Organization) growth norms at three and six months, and b) that have a z-score change <0.67 in weight-for-age WHO norms over time periods one to three months, and three to six months [27,88].

H2: The T4TM plus MIHP (intervention group) as compared to the MIHP only (control group) will have: a) higher average maternal responsiveness scores, b) greater probability of using a positive feeding style, and c) greater average delay in introducing solid foods in keeping with AAP guidelines.

## Methods/Design

### Overall study design

This randomized, experimental, short-term, longitudinal controlled trial uses a convenience sample of low-income adolescent first-time mothers of infants from four counties in Michigan. Participants are randomly assigned to the T4TM intervention or a control group with a retention goal of N = 40 participants per group by Time 3 data collection. Data are collected when infants are 4–8 weeks of age (Time 1), 10–12 weeks of age (Time 2), and 6 months of age (Time 3).

### Development of the intervention

The T4TM intervention was developed based on the Healthy Babies through Infant-Centered Feeding intervention curriculum [64], which provided evidence that education about infant-centered feeding had a positive impact on low-income mothers’ infant feeding knowledge and self-reported feeding behaviors. Behaviors targeted in the T4TM intervention were derived from theories and empirical studies of mother-infant interaction [86,89-91]. The infant-centered feeding experience comprises maternal responsiveness (MR), feeding styles (FS), and feeding practices (FP). Maternal responsiveness is maternal behavioral sensitivity to infant cues through accurate recognition and judgment of what the infant needs [91-97]. Feeding style pertains to the mother’s beliefs about and approach (authoritative, authoritarian, permissive, or uninvolved) to guiding her infant’s feeding behaviors [97-101]. Feeding practices are maternal behaviors relating to what, where, how, and how often a baby is fed [99]. Maternal behaviors affect infant growth over time [102-119].

Previous research by the Principal Investigators to inform the development of T4TM challenge content, activities, and design included focus groups in which adolescent mothers identified areas of interest and need related to infant-centered feeding practices [120]. Results from two focus groups with low-income, adolescent, first-time mothers of infants [121] (N = 16) revealed interests in: a) lessons on infant feeding, b) learning about the proper introduction of solid food, c) how much formula babies should be receiving, and d) learning to recognize hunger and satiation cues.

### Participants and recruitment

To be eligible, a potential participant must: be low-income (≤185% federal poverty level in the United States and eligible for MIHP services), be between 15 and 19 years of age at time of enrollment, be a first time mom, have an infant two months of age or less, be a primary caregiver who feeds her infant at least once a day, have text messaging and web access, and have had a term birth (37 ≤ 42 weeks, 2500 ≤ 3750 grams birth weight). If either mother or infant has been diagnosed with an eating or nutritional condition, the dyad is ineligible. Staff members from MIHP offices in four Michigan counties target mother-infant dyads, providing information about the study to recruit mothers with infants or expectant mothers to enroll in the project.

Once eligibility is established, families are contacted for the initial data collection home visit where written informed consent is obtained. We have institutional approval to conduct this trial from the University Committee on Research Involving Human Subjects from Michigan State University.

### Randomization

Data collection packets are pre-randomized into either T4TM or control group, keeping the assigned group blind to the data collectors until they arrive at participants’ homes to conduct the baseline data collection. Participants are randomly assigned via computer randomization procedure using Microsoft Excel, balancing groups by the four county recruitment sites.

### T4TM intervention

T4TM provides a new daily challenge over six weeks, a time frame selected to provide participants with enough opportunities to form the habit of visiting the T4TM web-application daily [122,123]. Participants log onto T4TM through their cell phones (or a computer), a technological requirement for the study that does not pose an undue burden on participants, especially as low-income households use social networks more frequently than adolescents in higher-income households [71,72]. Participants are encouraged to engage with T4TM as frequently as they like. Frequency of use and number of challenge completion will be logged by the application. Throughout the six-week intervention, participants will receive a daily text message, containing the challenge name and the URL to open the web-application directly through their smartphones. When the participants visit the T4TM page they will see: 1) the name of the challenge, 2) why the challenge is important, 3) tips to complete the challenge, 4) how many people have completed the daily challenge, and 5) a space to leave comments. In addition to the rotating challenges, adolescent mothers can browse T4TM for information about infant feeding, links to related topics, a discussion forum, and a place where they can receive expert advice from a RD or RN. When participants complete a daily challenge, they may click on the “I Did This” button to publicly acknowledge their accomplishment, which serves as a motivational feedback function as they observe other mothers’ completion of the challenges, thereby establishing healthy feeding practices as normative for the young mothers [124]. Participants continue to receive usual MIHP care during the intervention.

### Procedures

Data are obtained by trained data collectors using both self-report questionnaires and anthropometric assessment. To ensure consistency and accuracy of data collection, a half-day intensive training session was held for data collectors prior to the collection of data, to be followed by a booster session in year 2. Training consisted of a standardized training guide and protocol including review of the instruments and measurement of infant recumbent length and weight. Telephone contact and email communication occurs between the project manager and the data collector and/or supervisor to facilitate open communication and fidelity to the protocol.

Intervals for data collection reflect time points (at infant age of 4–8 weeks, 3 months, and 6 months old) that align with both infant feeding recommended milestones and the intervention duration. For example, the third data collection occurs when the infant is 6 months old, when solids foods introduction is recommended.

### Measures

#### Maternal outcomes

**Maternal responsiveness** is defined as sensitivity of the behaviors of a mother to her infant’s cues through expert judgment of what her infant needs [91-95]. These behaviors comprise a relationship skill set promoting mothers’ accurate recognition and response to infants’ feeding cues [96,97]. The Maternal Infant Responsiveness Instrument [125] is used to measure maternal responsiveness to infant cues; the scale is valid [126] and reliable (alpha = .83 and .87) [41,127]. We expect the intervention group to have higher scores than the control group.

**Feeding style** expresses the mother’s beliefs about and approach to guiding her infant’s feeding behaviors [97,98]. It also describes how (style) mothers feed their infants (authoritative, authoritarian, permissive, uninvolved) [99-101]. An adapted version of the Infant Feeding Styles Questionnaire (IFSQ) [128,129] is used to measure feeding beliefs and feeding style behaviors [42,43,97-99,129,130]. The IFSQ was pretested and validated with 150 African American first-time mothers with children ≤ 2 years of age [43]. Reliabilities for subscales range from .75 to.95 [128]. We expect the intervention group to score higher in the authoritative range than the control group.

**Feeding practices** are maternal behaviors relating to what is fed, where, how, and how often; they are essential for ensuring healthy eating habits throughout childhood [98,102-104]. The Infant Feeding Index (IFI) [131-134] identifies age-appropriate beverages and foods offered to the infant [135-138] and the appropriateness of feeding environment (i.e., support infant’s self-regulation) per AAP recommendations is a valid measure [131,133,135,136,139]. Of primary concern is the time at which solid foods are first introduced into the infant’s diet. We expect a longer average delay of introduction to solid foods that is closer to the AAP recommendation in the intervention group compared to the control group.

#### Infant outcome

**Infant growth** is the measurement of an infant’s weight and length plotted over time using *z* scores computed from the measures taken at T1 (baseline),T2, post intervention; and T3. Infant weights and lengths will be standardized as weight-for-age percentiles (WAP) and weight-for-age *z*-scores (WAZ), using WHO Anthro [140]. Relative weight gain (or loss) can then be calculated as the difference between WAZ scores at T3 and T1 [140]. Rate of growth proportional to birth weight and body composition (a weight-for-length index) will also be calculated. We expect a greater proportion of infants in the intervention group: 1a) will fall below the 85th percentile of the WHO growth norms at three and six months; and 1b) z score change <0.67 in weight-for-age WHO norms over time periods one-to-three months, and three-to-six months.

The infant’s weight and length is measured at all three time points to provide an objective measure of weight status. The infant is weighed in a clean diaper on an electronic scale and weighed to the nearest half ounce. Infant length is measured in the recumbent position. Digital scales are calibrated prior to weights being taken. These data will be entered into EpiInfo V3.4.3 to calculate weight-for-length z scores.

### Maternal and infant conditions that may influence outcomes

Maternal behaviors affect infant growth over time [102-119] and are the focus within the maternal-infant feeding interaction. Several background factors also affect infant growth [141,142], such as maternal knowledge [143], self-efficacy [144], and infant temperament. The Maternal Knowledge and Self-Efficacy scale [64], a 13-item knowledge scale and 7-item, 6-point Likert self-efficacy scale of dis/agreement, has been used with lower-income mothers of infants (alpha = .72 to .75) [64]. The standardized Pictorial Assessment of Temperament (PAT) [145], a 10-item tool that requires mothers to select how they would categorize their infants’ reaction to everyday situations as represented by drawings, is used for this study. The PAT provides a mean score ranging from 1 to 3 [145], and demonstrates convergent validity with other infant temperament questionnaires [146]. Demographic information is collected via a self-report questionnaire, including age, race, and maternal health.

### Feasibility variables

Feasibility is assessed by rates of enrollment (percent of eligible and approached adolescents), the percentages of daily challenges completed overall, and by individual participants. These data are monitored by project staff through website tracking protocols. Attrition dates and reasons are tracked on all participants enrolled in the study by the project manager. Acceptability and satisfaction are measured at time 2 data collection using a questionnaire developed by the research team that allows participants to rate the utility of the T4TM challenges and content, and give their opinions about the format, ease of use, and delivery method [64].

### Sample size and power calculations

Using non-intervention levels of infant growth to determine sample size, a power analysis using a time-averaged difference (TAD) method [147] indicated a sample size of 80 participants (40 per group) would be necessary to achieve power at .80, with a one-tailed TAD p < 0.05 over three measures and assuming a compound symmetry covariance structure with p = 0.20. A sample of 80 is deemed adequate; it is higher than the often recommended 12 per group [148]. The primary foci of this study include assessment of: 1) the effect size of the intervention to power a larger study, 2) feasibility, 3) practicality of data collection procedures, 4) measurement adequacy, and 5) the ability to implement.

### Data analysis

Outcomes (infant growth, MR, FS, FP) and maternal and infant factors at baseline (self-efficacy, knowledge, infant age, temperament, type of feeding) will be compared across intervention and control groups using t-test for continuous variables and chi-square test for categorical variables [149,150]. If differences are observed, the relevant variables will be treated as covariates in our post-experiment analyses [149,151,152]. All analyses will use the intention-to-treat [153] principle based on imputed data. The hypothesis regarding differences in outcomes at each follow-up T2 and T3 between intervention and control groups will be tested using the generalized estimating equation (GEE) model [154] for proportional group differences in growth over time. To explore efficacy of the intervention as assessed by MR, FS, FP, evaluated pre-intervention (T1), immediately post intervention (T2), and when the infant is six months old (T3). Both general linear mixed models (GLMM) [155,156], for continuous outcomes and the GEE model for logistic or Poisson regression for binary/ordinal and count outcomes will be considered.

## Discussion

Our long-term goal is to reduce the risk for infant obesity through an infant-centered feeding skill-building, educational social media intervention, which is accessible and suitable for adolescents. The T4TM study will provide: 1) increased knowledge about use of social media as a platform for helping adolescent mothers of infants develop healthy infant feeding practices that will contribute to the overall health and development of the infant; 2) specific statistical measurements of the feasibility of a web application; and 3) a research-based, empirically-tested obesity prevention curriculum product for use by community programs serving low-income adolescent mothers. Thus, the study should contribute to the scientific literature by generating new knowledge of the behavioral factors that influence childhood obesity. In addition, findings from this study will be used to enhance pre-existing education and community programs that target adolescent mothers.

In summary, T4TM is a theory-based, social media intervention that has the potential to be sustainable, given that it can be implemented in existing infrastructures delivered by community-based educators and health professionals. By providing T4TM in partnership with such agencies, the potential exists to enhance programming nationwide through broad-based dissemination and may thus have important implications for early childhood programs.

## Abbreviations

RWG: Rapid weight gain
MR: Maternal responsiveness
FS: Feeding styles
FP: Feeding practices
AAP: American Academy of Pediatrics
T4TM: Tools 4 Teen Moms
RCT: Randomized controlled trial
MIHP: Maternal Infant Health Program
RN: Registered Nurse
LSW: Licensed Social Worker
RD: Registered Dietician
WHO: World Health Organization
IFSQ: Infant Feeding Styles Questionnaire
IFI: Infant Feeding Index
WAP: Weight-for-age percentiles
WAZ: Weight-for-age *z*-scores
PAT: Pictorial Assessment of Temperament
TAD: Time-averaged difference
GEE: Generalized estimating equation
GLMM: General linear mixed models
